# *DBP* rs7041 and *DHCR7* rs3829251 are Linked to CD4^+^ Recovery in HIV Patients on Antiretroviral Therapy

**DOI:** 10.3389/fphar.2021.773848

**Published:** 2022-01-18

**Authors:** Salvador Resino, María Ángeles Jiménez-Sousa, Julià Blanco, Yolanda M. Pacheco, Jorge del Romero, Joaquim Peraire, Ana Virseda-Berdices, María José Muñoz-Gómez, Carlos Galera-Peñaranda, Lucio Jesus García-Fraile, José M. Benito, Norma Rallón

**Affiliations:** ^1^ Unidad de Infección Viral e Inmunidad, Centro Nacional de Microbiología, Instituto de Salud Carlos III, Majadahonda, Spain; ^2^ Centro de Investigación Biomédica en Red en Enfermedades Infecciosas, Instituto de Salud Carlos III, Madrid, Spain; ^3^ Virología e Inmunología Celular, IrsiCaixa AIDS Research Institute, Badalona, Spain; ^4^ Laboratorio de Inmunología, Instituto de Biomedicina de Sevilla (IBiS), Sevilla, Spain; ^5^ Centro Sanitario Sandoval, Hospital Clínico San Carlos, Madrid, Spain; ^6^ Departamento de Medicina Interna, Hospital Universitari de Tarragona Joan XXIII, Tarragona, Spain; ^7^ Departamento de Medicina Interna, Hospital Universitario Virgen de la Arrixaca, Murcia, Spain; ^8^ Departamento de Medicina Interna, Hospital Universitario de La Princesa, Madrid, Spain; ^9^ Laboratorio de Investigación Del VIH y la Hepatitis Viral, Instituto de Investigación Sanitaria Fundación Jiménez Díaz, Universidad Autónoma de Madrid (IIS-FJD, UAM), Madrid, Spain; ^10^ Hospital Universitario Rey Juan Carlos, Móstoles, Spain

**Keywords:** HIV, antiretroviral therapy, DHCR7, DBP, SNP, immune reconstitution, CD4^+^ T cells

## Abstract

**Background:** The lack of the recovery of CD4^+^ T-cells (CD4^+^ recovery) among immunodeficiency virus (HIV)-infected patients on antiretroviral therapy (ART) is not well known. We aimed to analyze the association between single nucleotide polymorphisms (SNPs) underlying vitamin D metabolism and the CD4^+^ recovery in naïve HIV-infected patients who started ART with low baseline CD4^+^.

**Methods:** We conducted a retrospective study in 411 naïve individuals with plasma HIV load >200 copies/mL and CD4^+^ <200 cells/mm^3^. During 24 months of follow-up, all patients had plasma HIV load <50 copies/mL. DNA genotyping was performed using the Sequenom MassARRAY platform. The outcome variable was the change in CD4^+^ during the study.

**Results:** CD4^+^ recovery was higher in patients carrying *DBP* rs7041 AA genotype (AA versus CC/AC) and *DHCR7* rs3829251 AA genotype (AA versus GG/AG) (*p*-value < 0.05). *DBP* rs7041 AA genotype was linked to increase in CD4^+^ (adjusted arithmetic mean ratio (aAMR) = 1.22; *q*-value = 0.011), increase in CD4^+^ ≥P75th [adjusted odds ratio (aOR) = 2.31; *q*-value = 0.005], slope of CD4^+^ recovery (aAMR = 1.25; *q*-value = 0.008), slope of CD4^+^ recovery ≥ P75th (aOR = 2.55; *q*-value = 0.005) and achievement of CD4^+^ ≥500 cells/mm^3^ (aOR = 1.89; *q*-value = 0.023). Besides, *DHCR7* rs3829251 AA genotype was related to increase in CD4^+^ (aAMR = 1.43; *q*-value = 0.031), increase in CD4^+^ ≥P75th (aOR = 3.92; *q*-value = 0.030), slope of CD4^+^ recovery (aAMR = 1.40; *q*-value = 0.036), slope of CD4^+^ recovery ≥ P75th (aOR = 3.42; *q*-value = 0.031) and achievement of CD4^+^ ≥500 cells/mm^3^ (aOR = 5.68; *q*-value = 0.015).

**Conclusion:** In summary, *DHCR7* rs3829251 and *DBP* rs7041 polymorphisms were associated with CD4^+^ recovery in HIV-infected patients who started cART with low CD4^+^ T-cell counts.

## Introduction

Combination antiretroviral therapy (cART) tends to achieve undetectable plasma viral load levels in the vast majority of the human immunodeficiency virus (HIV)-infected patients treated. This control of viral replication allows the recovery of CD4^+^ T-cells (CD4^+^ recovery) in peripheral blood, and many immune functions are restored (Panel de expertos de Gesi, 2011; Thompson et al., 2012). Despite this, there is still a significant percentage (around 30%) of HIV-infected patients who fail to have complete CD4^+^ recovery (≥500 CD4^+^ T‐cell count/µl) after long periods of cART (Yang et al., 2020). Those patients who maintain low CD4^+^ counts remain at risk of acquired immunodeficiency syndrome (AIDS) progression, developing non-AIDS-related morbidity, and dying (Baker et al., 2008; Kelley et al., 2009; Helleberg et al., 2013).

The causes of this lack of CD4^+^ recovery among cART-treated patients are not well known, but it appears to be a complex and multifactorial phenomenon (Yang et al., 2020). In this regard, many factors involved in CD4^+^ recovery have been described, among which include age, low CD4^+^ T-cells nadir, severe immunodeficiency at the time of cART initiation, low baseline CD4/CD8 ratio, immune exhaustion, abnormal immune activation, reduced output in the bone marrow and thymic, increased senescence and apoptosis of T-cells, lymphoid tissue fibrosis, imbalance in Treg and Th17 cells, microbial translocation, persistent HIV replication, and host genetic background, among others (Yang et al., 2020). However, all these factors do not fully explain the great variability of immune reconstitution in cART-treated patients.

Vitamin D (VitD) deficiency is common in HIV-infected patients (around 70–85%) (Mansueto et al., 2015; Gois et al., 2017). VitD regulates different antimicrobial pathways of immunity that can be crucial against HIV infection (Jiménez-Sousa et al., 2018). Besides, VitD deficiency has been related to higher HIV viral load values in plasma, inflammation, immune activation, decreased CD4^+^ T-cells, rapid AIDS progression in cART-naïve patients, and impaired CD4^+^ recovery in HIV-infected patients on cART (Jiménez-Sousa et al., 2018).

**FIGURE 1:**
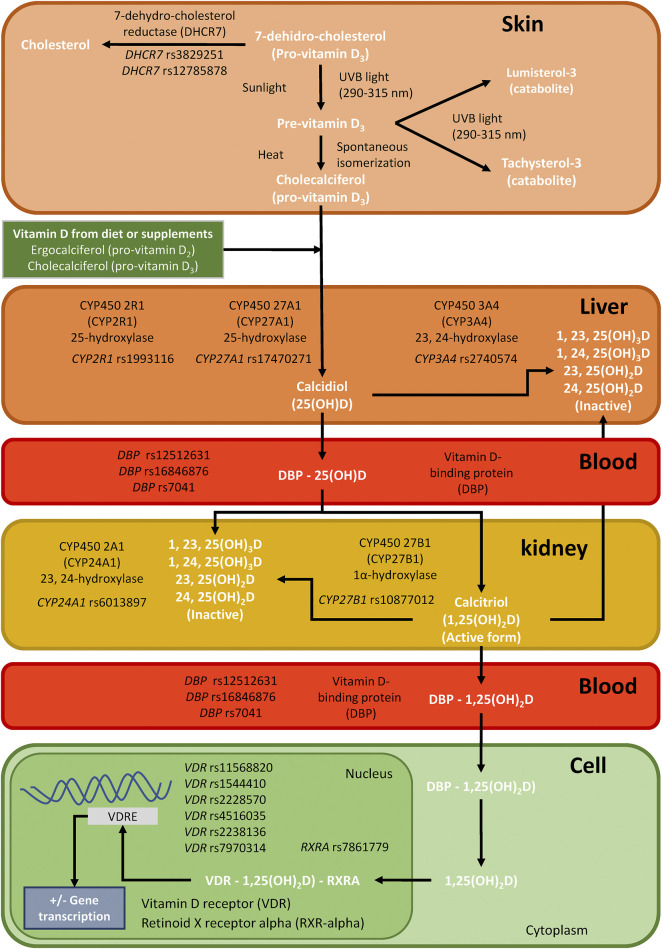
Summary of single nucleotide polymorphisms (SNPs) analyzed for this study in genes related to the synthesis, transport, and mechanism of action of vitamin D in the body.; Abbreviations: DHCR7, 7-dehydrocholesterol reductase; CYP2R1, cytochrome P450 enzymes; CYP27A1, cytochrome P450 enzymes; CYP27B1, cytochrome P450 enzymes; CYP3A4, cytochrome P450 enzymes; CYP24A1, cytochrome P450 enzymes DBP, vitamin D-binding protein; VDR, vitamin D receptor; and RXRA, retinoid X receptor alpha.

Most VitD is produced in the body, and only a small percentage is ingested in the diet. In the first step, pro-VitD (7-dehydro-cholesterol) is transported to the skin, where it is isomerized to pre-VitD (cholecalciferol) by ultraviolet irradiation (Herrmann et al., 2017). However, 7-dehydrocholesterol reductase (DHCR7) may oxidate 7-dehydro-cholesterol to cholesterol, decreasing the amount of 7-DHC available for photochemical conversion to VitD in the skin (Prabhu et al., 2016). Next, pre-VitD is hydroxylated to 25-hydroxy-VitD [25(OH)D] in the liver by cytochrome P450 enzymes (CYP27A1 and CYP2R1). Then, 25 (OH)D is transported to the kidneys by the vitamin D-binding protein (DBP), where the 25-hydroxyvitamin D_3_ 1-alpha-hydroxylase (CYP27B1) forms calcitriol [1,25 (OH)2D], which is the metabolically active form (Herrmann et al., 2017; Jiménez-Sousa et al., 2018). Besides, 25 (OH)D is catabolized by CYP3A4, and CYP24A1 catabolizes 1,25 (OH)2D. In the nucleus, VitD binds to vitamin D receptor (VDR) and promote the formation of a heterodimer with the retinoid X receptor alpha (RXRA), which binds to vitamin D response elements (VDRE), initiating the transcription of more than 4,000 genes (around 5% of the human genome) (Herrmann et al., 2017; Jiménez-Sousa et al., 2018) (Figure 1).

Several single nucleotide polymorphisms (SNPs) in genes related to VitD metabolism (*DHCR7*, *CYP2R1*, *CYP27A1*, *CYP27B1*, *CYP3A4*, *CYP24A1*, *DBP*, *VDR*, and *RXRA*, among others) have been associated with plasma levels of 25(OH)D and infectious diseases (Jolliffe et al., 2016). In HIV infection, *VDR* SNPs have been related to protection against HIV infection (de la Torre et al., 2008; Alagarasu et al., 2009; Torres et al., 2010). Also, *DHCR7, DBP*, *VDR,* and *CYP27B1* SNPs were associated with AIDS progression in ART-naïve HIV-infected patients of European origin (Eales et al., 1987; Nieto et al., 2004; Moodley et al., 2013; Laplana et al., 2014; Jiménez-Sousa et al., 2019a; Jiménez-Sousa et al., 2019b; Jiménez-Sousa et al., 2020). However, there is no data about the relationship between these SNPs in genes related to VitD metabolism and immune recovery in patients on cART.

## Objective

We aimed to analyze the association between genetic variants underlying VitD metabolism (*DHCR7, CYP2R1*, *CYP27A1*, *CYP27B1*, *CYP3A4, CYP24A1, DBP*, *VDR,* and *RXRA* genes) and the CD4^+^ recovery in naïve HIV-infected patients who started cART with low baseline CD4^+^ T cells (<200 cells/mm^3^).

## Material and Methods

### Study Population

We carried out a retrospective study in 411 naïve HIV-infected patients who started cART, included in two cohorts, the Spanish AIDS Research Network cohort (CoRIS, see Appendix 1) and the AIDS Research Institute IrsiCaixa-HIVACAT cohort. This cohort study has been previously described (Restrepo et al., 2019). Patients signed informed consent before participating in the study. The Ethics Committee of the “Fundación Jiménez Díaz” approved the research project (Ref.: PIC 52/2015_FJD). This study was performed under the Declaration of Helsinki.

The inclusion criteria were: 1) at baseline: naïve-ART, plasma HIV-RNA >200 copies/mL, CD4^+^ <200 cells/mm^3^, and DNA sample available; 2) during the follow-up period (2 years): plasma HIV-RNA <50 copies/mL, periodic monitoring of CD4 and plasma HIV-RNA**.** The two cohorts totaled 6,160 HIV-infected patients, of which 4,000 started cART, but only 1,259 had CD4^+^ <200 cells/mm^3^ before cART initiation. Of them, only 503 patients had a minimum follow-up period of 24 months, but 92 patients had no undetectable viral load (VL, HIV-RNA <50 copies/mL) after cART or DNA sample available. Finally, 411 patients met all the inclusion criteria. Besides, 145 healthy donors from the “Centro de Transfusión de la Comunidad de Madrid” were used as Control-group (HIV, HCV, and HBV seronegative subjects).

### Clinical Data

Demographic, clinical, virological, and laboratory data were collected from medical records. Gender was by self-identification and there were no transgender people. Time since HIV diagnosis was calculated from the first positive blood test for HIV. The mode of transmission (injecting drug use and sexual behavior) was inferred from the medical record. Hepatitis C and hepatitis B coinfection were determined by a standard laboratory test. The clinic management of patients was carried out following national clinical guidelines (Panel de expertos de Gesi, 2011).

### DNA Genotyping

Blood samples were collected by venipuncture in EDTA tubes. The blood samples were then sent to the HIV HGM BioBank (http://hivhgmbiobank.com/?lang=en), where the samples were processed and frozen immediately upon receipt. DNA isolation was performed from peripheral blood mononuclear cells using a QIAamp DNA kit (Qiagen, Spain).

We selected 17 SNPs of VitD metabolism [*DHCR7* (rs3829251 and rs12785878), *CYP2R1* (rs1993116), *CYP27A1* (rs17470271), *CYP27B1* (rs10877012), *CYP3A4* (rs2740574), *CYP24A1* (rs6013897), *DBP* (rs12512631, rs16846876, and rs7041), *VDR* (rs11568820, rs1544410, rs2228570, rs4516035, rs2238136, rs7970314), and *RXRA* (rs7861779)] (Figure 1), which have been related to circulating concentrations of VitD and non-skeletal diseases (Jolliffe et al., 2016). DNA genotyping was performed using the iPLEX® Gold technology and Agena Bioscience’s MassARRAY platform (San Diego, CA, United States) in the Spanish National Genotyping Center (CeGen; http://www.cegen.org/). All SNPs had a DNA genotyping success rate greater than 95%.

The validation and quality control of the genotyping process was performed using: 1) Negative controls, no template controls (NTC). The NTCs were used to confirm that no artifacts associated with design or chemistry were generated during the genotyping assays; 2) Positive controls. As positive controls, a trio of Coriell samples from the Human Genetic Cell Repository (NA10861, NA11994, and NA11995) was included in each genotyping assay. These Coriell samples were included in the set of genotyped samples in 1000GENOMES_phase_3 (EUR), so we could confirm the concordance of our results with those obtained for the 1000 Genomes Project. During the genotyping assays, we have replicated more than 18% of obtained genotypes, and we have observed a total concordance among replicated samples. Additionally, a phenotype-blind genotyping process was followed, since all patients who met the inclusion criteria were genotyped anonymously without information on their phenotype.

### Outcome Variables

Outcome variables were related to changes in CD4^+^ values during the 24 months of the study. The outcome variables analyzed were: 1) increases in CD4^+^ (ΔCD4^+^, continuous), which is the difference between the baseline and end of follow-up (month 24). 2) increases in CD4^+^ ≥P75th (dichotomous). 3) slope or gradient of CD4^+^ recovery (continuous), which is the ratio between the change in CD4^+^ and the time elapsed. 4) slope of CD4^+^ recovery ≥ P75th (dichotomous). 5) achieving CD4^+^ at the end of follow-up ≥500 cells/mm^3^ (dichotomous).

### Statistical Analysis

Both SPSS 22.0 (IBM Corp., Chicago, United States) and Stata 15.0 (StataCorp, Texas, United States) were used to carry out the statistical analysis. *P*-values < 0.05 were considered significant, and all tests were two-tailed.

For the descriptive study, the Chi-squared test or Fisher´s exact tests were used to compare categorical data and evaluate the Hardy-Weinberg equilibrium (HWE). Mann-Whitney U test and Kruskal-Wallis tests were used to compare continuous variables. The genetic association study between SNPs and clinical outcomes was assessed according to dominant, recessive, and additive models by Generalized Linear Models (GLMs). Specifically, we used a GLM with a gamma distribution (log-link) for continuous variables and a GLM with a binomial distribution (logit-link) for dichotomous variables, which reported the arithmetic mean ratio (AMR), and the odds ratio (OR), respectively. Additionally, the raw p-values were corrected using the false discovery rate (FDR) with Benjamini and Hochberg (*q*-values), a widely used multiple comparison adjustment method. The selected SNPs with *q*-values < 0.05 were evaluated by multivariate regression using GLM models adjusted by the main clinical characteristics at baseline: age, gender, Caucasian origin, hepatitis C and hepatitis B coinfection, HIV transmission by intravenous drugs use (IDU), cART regimen with protease inhibitors (PI), time since HIV diagnosis, and baseline CD4^+^ T cell count.

In addition, pairwise linkage disequilibrium (LD) analysis was computed by Haploview 4.2 software (Barrett et al., 2005). Haplotype-based association testing was performed using the PLINK package (Purcell et al., 2007).

## Results

### Population Characteristics

Table 1 shows the baseline characteristics of HIV-infected patients, whose median age was 40 years, around 79% were male, and 13% were coinfected with HCV or HBV. All patients were naïve ART and had CD4^+^ <200 cells/mm^3^ and detectable plasma HIV-RNA.

**TABLE 1:** Clinical and epidemiological characteristics at baseline of HIV infected patients who started cART with very low CD4+T-cells count (<200 cells/mm^3^).

Characteristics	Values
*n*	411
Male (*n* = 411) (%)	323 (78.6%)
Age (*n* = 411) (years)	40 34; 48)
Coinfections (*n* = 411) (%)	
Hepatitis C infection	32 (7.8%)
Hepatitis B infection	20 (4.9%)
Caucasian origin (*n* = 394) (%)	317 (80.5%)
Time since HIV diagnosis (*n* = 411) (years)	1 (1; 1)
CD4^+^ cell count (*n* = 411) (cells/mm^3^)	104 (41; 159)
cART regimen (*n* = 411) (%)	
PI-based	127 (31%)
NNRTI-based	205 (50%)
PI + NNRTI-based	53 (12.9%)
Others	25 (6.1%)
HIV transmission route (*n* = 384) (%)	
Homosexual transmission	189 (49.2%)
Heterosexual transmission	139 (36.2%)
IDU	56 (14.6%)

Statistical: Values were expressed as absolute number (percentage) and median (percentile 25; percentile 75), which were calculated with respect to the available data (in parentheses).

HIV, human immunodeficiency virus; cART, combination antiretroviral therapy; PI, HIV protease inhibitor; NNRTI, non-nucleoside analogue HIV reverse transcriptase inhibitor; IDU, intravenous drug users.

### Distribution of Genetic Single Nucleotide Polymorphisms

The distribution of SNPs related to the VitD pathway in healthy controls and HIV-infected patients is shown in Supplementary Table S1. All SNPs had values for minor allelic frequency (MAF) higher than 5%, and they were in HWE (*q*-value > 0.05). Healthy controls and HIV-infected patients had similar genotypic frequencies. All SNPs’ genotypic and allelic frequencies were in line with the NCBI SNP database for the European population (http://www.ncbi.nlm.nih.gov/projects/SNP/).

We also analyzed the LD between SNPs of the same gene (Supplementary Figure S1), finding very high LD values (D’ = 1.0) for *VDR* SNPs (rs4516035, rs11568820, and rs7970314) and *DHCR7* SNPs (rs12785878 and rs3829251). However, *r* ^2^ values were low for all SNPs (except rs11568820 vs rs7970314), indicating that each SNP provides different information.

### Association Between SNPs and CD4^+^ Recovery

Supplementary Table S2 shows the association between SNPs and CD4^+^ recovery by unadjusted GLMs. Overall, after correcting the p-values for multiple testing (FDR - Benjamini–Hochberg procedure), CD4^+^ recovery was higher in patients carrying *DBP* rs7041 AA genotype (AA versus CC/AC; recessive model) and *DHCR7* rs3829251 AA genotype (AA versus GG/AG; recessive model) (*q*-value <0.05).

**FIGURE 2:**
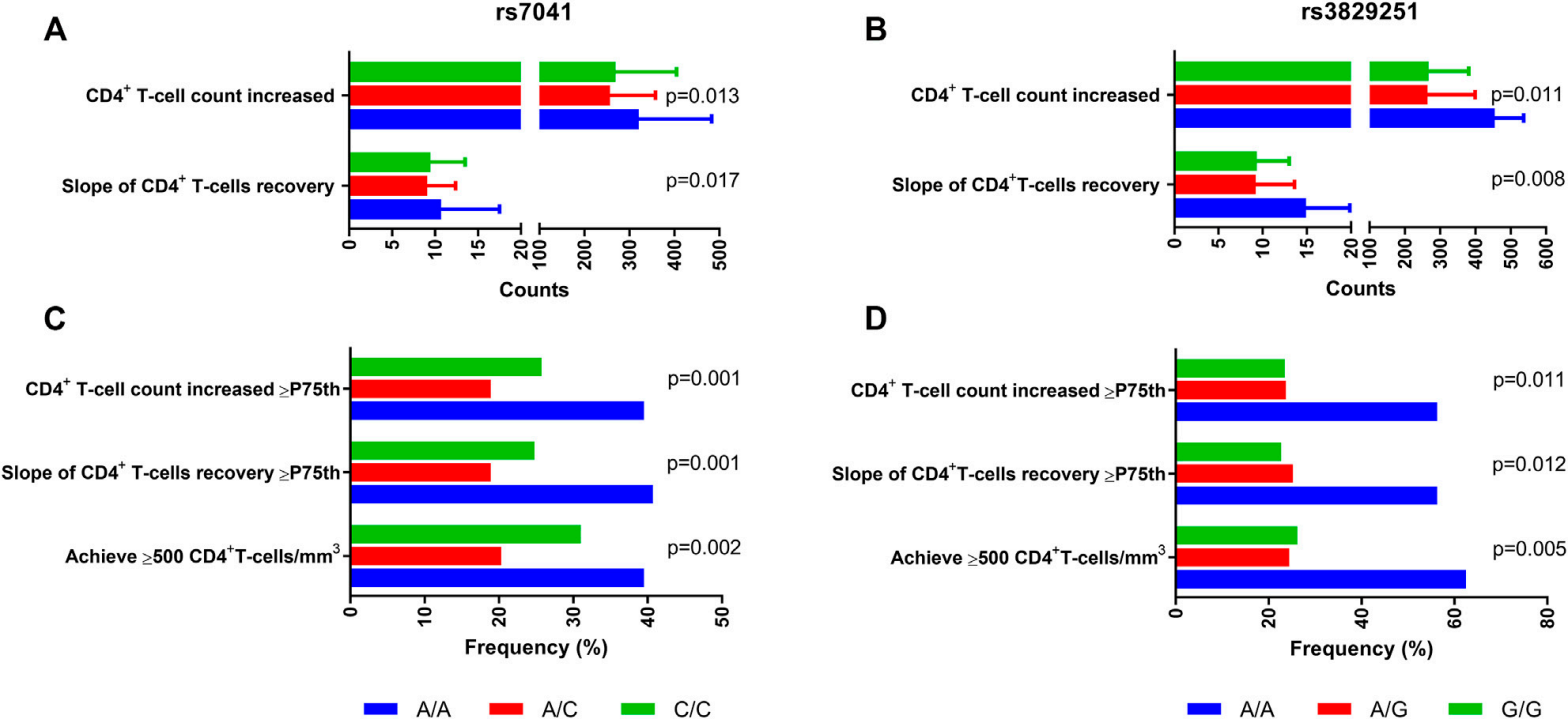
Summary of outcome variables of CD4^+^ recovery in HIV-infected patients who started ART with very low CD4^+^ T-cells count (<200 cells/mm^3^) according to *DBP* rs7041 and *DHCR7* rs3829251 polymorphisms. Statistics: Values were expressed as percentages and median (percentile 25; percentile 75). P-values were calculated by Chi-square and Kruskal-Wallis Tests.; Abbreviations: DBP, vitamin D binding protein; DHCR7, 7-Dehidrocolesterol reductase; HIV, human immunodeficiency virus.

Specifically, *DBP* rs7041 AA and *DHCR7* rs3829251 AA carriers had higher values of CD4^+^ T-cell count increased (*p*-value = 0.013 (Figure 2A) and *p*-value = 0.011 (Figure 2B), respectively), CD4^+^ T-cell count increased ≥ P75th (*p*-value = 0.001 (Figure 2C) and *p*-value = 0.011 (Figure 2D), respectively), slope of CD4^+^ recovery (*p*-value = 0.017 (Figure 2A) and *p*-value = 0.008 (Figure 2B), respectively), slope of CD4^+^ recovery ≥ P75th (*p*-value = 0.001 (Figure 2C) and *p*-value = 0.012 (Figure 2D), respectively), and percentage of patients achieving CD4^+^ ≥500 cells/mm^3^ (*p*-value = 0.002 (Figure 2C) and *p*-value = 0.005 (Figure 2D), respectively) than patients with other genotypes.

Then, the association of *DBP* rs7041 and *DHCR7* rs3829251 polymorphisms with CD4^+^ recovery was evaluated by adjusted GLMs (Table 2). *DBP* rs7041 AA genotype was linked to increase in CD4^+^ (adjusted arithmetic mean ratio (aAMR) = 1.22; *q*-value = 0.011), increase in CD4^+^ ≥P75th (adjusted odds ratio (aOR) = 2.31; *q*-value = 0.005), slope of CD4^+^ recovery (aAMR = 1.25; *q*-value = 0.008), slope of CD4^+^ recovery ≥ P75th (aOR = 2.55; *q*-value = 0.005) and achievement of CD4^+^ ≥500 cells/mm^3^ (aOR = 1.89; *q*-value = 0.023). Besides, *DHCR7* rs3829251 AA genotype was related to increase in CD4^+^ (aAMR = 1.43; *q*-value = 0.031), increase in CD4^+^ ≥P75th (aOR = 3.92; *q*-value = 0.030), slope of CD4^+^ recovery (aAMR = 1.40; *q*-value = 0.036), slope of CD4^+^ recovery ≥ P75th (aOR = 3.42; *q*-value = 0.031) and achievement of CD4^+^ ≥500 cells/mm^3^ (aOR = 5.68; *q*-value = 0.015).

**TABLE 2:** Association of *DBP* rs7041 and *DHCR7* rs3829251 single nucleotide polymorphisms with CD4^+^ T-cells recovery under a recessive inheritance model in HIV-infected patients who started ART with very low CD4+T-cells count (<200 cells/mm^3^).

Outcome variables	*DBP* rs7041 AA genotype			*DHCR7* rs3829251 AA genotype		
	Exp(b)	95%CI	*p*	Exp(b)	95%CI	*p*
CD4^+^ T-cell count increased	1.22	(1.05; 1.42)	**0.009**	1.43	(1.05; 1.95)	**0.023**
CD4^+^ T-cell count increased ≥ P75^th^	2.31	(1.34; 3.95)	**0.002**	3.92	(1.35; 11.39)	**0.012**
Slope of CD4^+^ T-cells recovery	1.25	(1.06; 1.45)	**0.005**	1.40	(1.02; 1.92)	**0.036**
Slope of CD4^+^ cells recovery ≥ P75^th^	2.55	(1.47; 4.41)	**0.001**	3.42	(1.17; 9.96)	**0.025**
Achieve ≥500 CD4^+^T-cells/mm^3^	1.89	(1.09; 3.28)	**0.023**	5.68	(1.81; 17.76)	**0.003**

Statistical: Values were calculated by multivariate regressions adjusted by the most important clinical and epidemiological characteristics (see statistical analysis section). Significant values are shown in bold.

Exp(b), exponentiation of the beta coefficient, which was arithmetic mean ratio (AMR) for continuous variables and odds ratio (OR) for categorical variables; DBP, vitamin D-binding protein; DHCR7, 7-Dehidrocolesterol reductase; HIV, human immunodeficiency virus; 95%CI, 95% of confidence interval; p, level of significance.

### Association Between Haplotypes and CD4^+^ Recovery

Supplementary Table S3 shows the association of *VDR*, *DBP*, *DHCR7* haplotypes with CD4^+^ recovery. We found some significant associations for the three genes and three dichotomic outcomes analyzed (*p*-value < 0.05), but these disappeared after FDR adjustment (*q*-value > 0.05).

## Discussion

Genetic background variability may confer differences in CD4^+^recovery in cART-treated HIV-infected patients (Guzmán-Fulgencio et al., 2013; Hartling et al., 2014; Yong et al., 2016; Hartling et al., 2017; Masson et al., 2018; Medrano et al., 2018; García et al., 2019). Our study found HIV-infected patients carrying the *DBP* rs7041 AA and *DHCR7* rs3829251 AA genotypes had a better CD4^+^ recovery after starting cART with low CD4^+^ T-cells count (<200 cells/mm^3^). We analyzed five outcome variables related to CD4^+^ recovery, and we found that rs7041 and rs3829251 were significantly associated with all outcome variables, which seems to indicate a clear impact on immune reconstitution. To our knowledge, this is the first time that these two SNPs (*DBP* rs7041 AA and *DHCR7* rs3829251 AA) have been linked to CD4^+^ recovery.

Multiple mechanisms have been reported by which VitD could influence the immune system (Jiménez-Sousa et al., 2018). VitD induces antiviral response against HIV by promoting antiviral gene expression, reducing the CCR5 expression on CD4^+^ T-cells, promoting an HIV-1-restrictive CD4^+^HLA-DR^-^ phenotype, and decreasing the impact of TNF-α in upregulating HIV replication in latently infected CD4^+^ T-cells (Aguilar-Jimenez et al., 2016; Nunnari et al., 2016). Interestingly, *DBP* rs7041 and *DHCR7* rs3829251 polymorphisms contribute to variation in plasma VitD level (Ahn et al., 2010; Lu et al., 2012), which has also been related to CD4^+^recovery (Jiménez-Sousa et al., 2018). Therefore, *DBP* rs7041 and *DHCR7* rs3829251 polymorphisms may be genetic variants to be further explored to increase our current knowledge of the mechanistic pathways involved in the poor immune recovery status and, also, assessing them as a potential target for improving immune reconstitution and prevent disease progression and death.

The *DBP* is a highly polymorphic gene that contains 13 exons and 12 introns and encodes an α2-glycosylated globulin. *DBP* SNPs have been associated with plasma VitD levels, affecting the function of VitD (Jolliffe et al., 2016). *DBP* rs7041 polymorphism is a missense variation of GAT→GAG, which changes aspartic acid at position 416 to glutamic acid. Rs7041 has been implicated in the generation of different DBP isoforms with different affinity for VitD metabolites (Speeckaert et al., 2006; Sinotte et al., 2009), which seem to affect the VitD delivery in the cell (Hibler et al., 2012). Additionally, *DBP* rs7041 polymorphism is related to the pathogenesis of various infectious diseases, such as coronavirus disease 2019 (COVID-19) (Karcioglu Batur and Hekim, 2020), susceptibility to HCV infection (Xie et al., 2018), chronic hepatitis C progression (Petta et al., 2013; Azevedo et al., 2017), respiratory syncytial virus bronchiolitis (Randolph et al., 2014) and AIDS progression in ART-naïve HIV-infected patients (Eales et al., 1987), as well as the response to antiviral therapy in HCV-infected patients (Falleti et al., 2012). However, the association with the cART response in HIV-infected patients has not yet been described. In our study, we found a positive impact of *DBP* rs7041 AA genotype on CD4^+^ recovery in naïve patients who started cART, but due to the high variability of the DBP protein (Jolliffe et al., 2016), we do not rule out that other *DBP* SNPs may be involved in the observed effect on CD4^+^ recovery.

The *DHCR7* gene encodes 7-dehydrocholesterol reductase that catalyzes the transformation of pro-VitD (7-dehydro-cholesterol) into cholesterol, acting as a switch between cholesterol and vitamin D synthesis (Prabhu et al., 2016). Regarding the genetic factors, several studies have reported that *DHCR7* rs3829251 contributes to variation in plasma VitD levels (Ahn et al., 2010; Wang et al., 2010; Lu et al., 2012), although its functional role is still unknown. Rs3829251 SNP is located within an intronic region of the *NADSYN1* gene and upstream of the *DHCR7* gene. Using the rVarBase database (Guo et al., 2016), we observed that rs3829251 SNP is implicated in changes in the chromatin state in different cell lines and tissues. These chromatin modifications can affect the DNA accessibility to transcription factors and thus, contribute to changes in the expression of both *NADSYN1* and *DHCR7* genes. In fact, rs3829251 has been associated with the *DHCR7* expression in the literature (Strawbridge et al., 2014). It seems to have a regulatory role on *NADSYN1* gene as expression and to splice quantitative trait loci (eQTL and sQTL), as described in numerous tissues by the Genotype-Tissue Expression Portal (GTEx Portal, https://gtexportal.org). Additionally, it is also important to note that a large number of SNPs between the nicotinamide adenine dinucleotide (NAD) synthetase‐1 (*NADSYN1*) gene and *DHCR7* gene (*NADSYN1/DHCR7* locus) are in high linkage disequilibrium (LD). Thus, we cannot rule out that other SNPs in high LD with rs3829251 SNP could be the causal polymorphism. Further studies would be needed to corroborate its functional role.

All newly diagnosed HIV-infected patients should initiate cART, regardless of CD4^+^ T cell count, to decrease the risk of HIV transmission and prevent the progression of AIDS and the occurrence of AIDS-related events (Saag et al., 2018). Late presentation to HIV care is a significant and persistent problem worldwide (Kranzer et al., 2012; Suárez-García et al., 2016; Croxford et al., 2018), even in developed countries with good healthcare access (Croxford et al., 2018). Due to the delay in the diagnosis of HIV infection, late presenters are a significant group of patients (Mocroft et al., 2013; Darling et al., 2016), who tend to have CD4^+^ T cell below 200 cells/mm^3^ in many cases and start cART late (Antinori et al., 2011), and thus, having worse CD4^+^ recovery rates (Negredo et al., 2010; Pérez-Molina et al., 2012).

Many reports have shown the inability to have a CD4^+^ recovery after long periods of cART, evaluating the outcomes in terms of immunological response and disease progression (Yang et al., 2020). There is no consensus on the definition of incomplete immune reconstitution. Our study analyzed the threshold for CD4^+^ T‐cell count >500/µL, which is one of the most accepted as an adequate immune response to cART since HIV‐1‐infected patients with >500 CD4^+^ T‐cells/µl have morbidity and mortality rates similar to those of HIV-negative people (Yang et al., 2020). Moreover, we also analyzed other immunological outcomes that indicate efficient CD4^+^ recovery but have a difficult clinical interpretation. However, we have not evaluated the relationship between poor immune recovery and clinical outcomes.

### Strengths and Limitations of the Study

Strengths: 1) We studied a very representative sample of the Spanish population infected with HIV because our cohort comes from a large number of hospitals spread throughout Spain. 2) We analyzed patients who had a baseline CD4^+^ T-cells <200 cells/mm^3^ and undetectable viral load during the whole follow-up period, strict criteria that help better define profiles of patients who recover and do not recover CD4^+^ T-cells. 3) The study period was the same in all patients (24 months after starting cART). 4) We evaluated different threshold values of CD4^+^ recovery that allow greater certainty when confirming the statistical association with CD4^+^ recovery.

Limitations: 1) Selection bias due to retrospective design and restrictive inclusion criteria. 2) Low statistical power due to the relatively small sample size, which may have affected the detection of less strong associations. 3) Our study was mostly performed on Caucasian individuals and more studies should be done in other populations. 4) Genotyping of more polymorphisms within genes involved in the VitD metabolim could provide additional insight into CD4^+^ T-cells recovery. 5) We have not performed functional assays to confirm the effect of *DHCR7* rs3829251 and *DBP* rs7041 polymorphisms on CD4^+^ T-cells recovery.

## Conclusion

In summary, *DHCR7* rs3829251 and *DBP* rs7041 polymorphisms were associated with CD4^+^ recovery in HIV-infected patients who started cART with low CD4^+^ T-cell counts. These SNPs in the VitD pathway could help detect HIV-infected patients with lower likelihood of CD4^+^ recovery after cART. However, further studies with more polymorphisms, in different ethnicities, and with larger samples are needed about the role of VitD genetic variants on CD4^+^ recovery in late presenters initiating cART.

## Data Availability

The datasets presented in this study can be found in online repositories. The names of the repository/repositories and accession number(s) can be found below: (https://data.mendeley.com/datasets/6pf269zztf/1).
