# Supplementary material for: DBP rs7041 and DHCR7 rs3829251 are Linked to CD4+ Recovery in HIV Patients on Antiretroviral Therapy
Source: Front Pharmacol. 2022 Jan 18;12:773848. doi: 10.3389/fphar.2021.773848 (PMC8804497; doi:10.3389/fphar.2021.773848)
Supplement: Supplementary file 1 [file DataSheet2.docx]

## **Appendix 1.**

**“Centers and investigators involved in CoRIS”**

**Executive committee**: Santiago Moreno, Inma Jarrín, David Dalmau, Maria Luisa Navarro, Maria Isabel González, Jose Luis Blanco, Federico Garcia, Rafael Rubio, Jose Antonio Iribarren, Félix Gutiérrez, Francesc Vidal, Juan Berenguer, Juan González.

**Fieldwork, data management and analysis**: Inma Jarrín, Belén Alejos, Victoria Hernando, Cristina Moreno, Carlos Iniesta, Luis Miguel Garcia Sousa, Nieves Sanz Perez

**HIV BioBanK:** Hospital General Universitario Gregorio Marañón: M Ángeles Muñoz-Fernández, Isabel María García-Merino, Irene Consuegra Fernández, Coral Gómez Rico, Jorge Gallego de la Fuente, Paula Palau Concejo.

**Participating centres:**

**Hospital General Universitario de Alicante (Alicante):** Joaquín Portilla, Esperanza Merino, Sergio Reus, Vicente Boix, Livia Giner, Carmen Gadea, Irene Portilla, María Pampliega, Marcos Díez, Juan Carlos Rodríguez, José Sánchez-Payá.

**Hospital Universitario de Canarias (San Cristobal de la Laguna):** Juan Luis Gómez, Jehovana Hernández, María Remedios Alemán, María del Mar Alonso, María Inmaculada Hernández, Felicitas Díaz-Flores, Dácil García, Ricardo Pelazas, Ana López Lirola.

**Hospital Universitario Central de Asturias (Oviedo):** Víctor Asensi, Eulalia Valle, María Eugenia Rivas Carmenado, Tomas Suarez-Zarracina Secades, Laura Pérez Is.

**Hospital Universitario 12 de Octubre (Madrid):** Rafael Rubio, Federico Pulido, Otilia Bisbal, Asunción Hernando, Lourdes Domínguez, David Rial Crestelo, Laura Bermejo, Mireia Santacreu.

**Hospital Universitario de Donostia (Donostia-San Sebastián):** José Antonio Iribarren, Julio Arrizabalaga, María José Aramburu, Xabier Camino, Francisco Rodríguez-Arrondo, Miguel Ángel von Wichmann, Lidia Pascual Tomé, Miguel Ángel Goenaga, Mª Jesús Bustinduy, Harkaitz Azkune , Maialen Ibarguren, Aitziber Lizardi, Xabier Kortajarena.

**Hospital General Universitario De Elche (Elche):** Félix Gutiérrez, Mar Masiá, Sergio Padilla, Andrés Navarro, Fernando Montolio, Catalina Robledano, Joan Gregori Colomé, Araceli Adsuar, Rafael Pascual, Marta Fernández, Elena García., José Alberto García, Xavier Barber.

**Hospital General Universitario Gregorio Marañón (Madrid):** Juan Berenguer, Juan Carlos López Bernaldo de Quirós, Isabel Gutiérrez, Margarita Ramírez, Belén Padilla, Paloma Gijón, Teresa Aldamiz-Echevarría, Francisco Tejerina, Francisco José Parras, Pascual Balsalobre, Cristina Diez, Leire Pérez Latorre.

**Hospital Universitari de Tarragona Joan XXIII (Tarragona):** Francesc Vidal, Joaquín Peraire, Consuelo Viladés, Sergio Veloso, Montserrat Vargas, Montserrat Olona, Anna Rull, Verónica Alba, Elena Yeregui, Jenifer Masip, Laia Reverté.

**Hospital Universitario y Politécnico de La Fe (Valencia):** Marta Montero Alonso, José López Aldeguer, Marino Blanes Juliá, María Tasias Pitarch, Iván Castro Hernández, Eva Calabuig Muñoz, Sandra Cuéllar Tovar, Miguel Salavert Lletí, Juan Fernández Navarro.

**Hospital Universitario La Paz/IdiPAZ:**  Juan González-garcia, Francisco Arnalich, José Ramón Arribas, Jose Ignacio Bernardino de la Serna, Juan Miguel Castro, Luis Escosa, Pedro Herranz, Victor Hontañón, Silvia García-Bujalance, Milagros García López-Hortelano, Alicia González-Baeza, Maria Luz Martín-Carbonero, Mario Mayoral, Maria Jose Mellado, Rafael Esteban Micán, Rocio Montejano, María Luisa Montes, Victoria Moreno, Ignacio Pérez-Valero, Berta Rodés, Talia Sainz, Elena Sendagorta, Natalia Stella Alcáriz, Eulalia Valencia.

**Hospital San Pedro Centro de Investigación Biomédica de La Rioja (CIBIR) (Logroño):** José Ramón Blanco, José Antonio Oteo, Valvanera Ibarra, Luis Metola, Mercedes Sanz, Laura Pérez-Martínez.

**Hospital Universitari MutuaTerrassa (Terrasa):** David Dalmau, Angels Jaén, Montse Sanmartí, Mireia Cairó, Javier Martinez-Lacasa, Pablo Velli, Roser Font, Mariona Xercavins, Noemí Alonso.

**Complejo Hospitalario de Navarra (Pamplona)** María Rivero, Jesús Repáraz, María Gracia Ruiz de Alda, María Teresa de León Cano, Beatriz Pierola Ruiz de Galarreta.

**Corporació Sanitària Parc Taulí (Sabadell):** Ferrán Segura, María José Amengual, Gemma Navarro, Montserrat Sala, Manuel Cervantes, Valentín Pineda, Sonia Calzado, Marta Navarro.

**Hospital Universitario de La Princesa (Madrid):** Ignacio de los Santos, Jesús Sanz Sanz, Ana Salas Aparicio, Cristina Sarriá Cepeda, Lucio Garcia-Fraile Fraile, Enrique Martín Gayo.

**Hospital Universitario Ramón y Cajal (Madrid):** Santiago Moreno, José Luis Casado, Fernando Dronda, Ana Moreno, María Jesús Pérez Elías, Cristina Gómez Ayerbe, Carolina Gutiérrez, Nadia Madrid, Santos del Campo Terrón, Paloma Martí, Uxua Ansa, Sergio Serrano, María Jesús Vivancos.

**Hospital General Universitario Reina Sofía (Murcia)** Enrique Bernal, Alfredo Cano , Antonia Alcaraz García, Joaquín Bravo Urbieta, Ángeles Muñoz, Maria Jose Alcaraz, Maria del Carmen Villalba**.**

**Hospital Nuevo San Cecilio (Granada):** Federico García, José Hernández, Alejandro Peña, Leopoldo Muñoz, Paz Casas, Marta Alvarez, Natalia Chueca, David Vinuesa, Clara Martinez-Montes.

**Centro Sanitario Sandoval (Madrid):** Jorge Del Romero, Carmen Rodríguez, Teresa Puerta, Juan Carlos Carrió, Mar Vera, Juan Ballesteros, Oskar Ayerdi.

**Hospital Universitario Son Espases (Palma de Mallorca):** Melchor Riera, María Peñaranda, María Leyes, Mª Angels Ribas, Antoni A Campins, Carmen Vidal, Francisco Fanjul, Javier Murillas, Francisco Homar.

**Hospital Universitario Virgen de la Victoria (Málaga):** Jesús Santos, Crisitina Gómez Ayerbe, Isabel Viciana, Rosario Palacios, Carmen María González.

**Hospital Universitario Virgen del Rocío (Sevilla):** Pompeyo Viciana, Nuria Espinosa , Luis Fernando López-Cortés**.**

**Hospital Universitario de Bellvitge (Hospitalet de Llobregat):** Daniel Podzamczer, Elena Ferrer, Arkaitz Imaz, Juan Tiraboschi, Ana Silva, María Saumoy.

**Hospital Costa del Sol (Marbella):** Julián Olalla, Alfonso del Arco, Javier de la torre, José Luis Prada, José María García de Lomas Guerrero, Javier Pérez Stachowski.

**Hospital General Universitario Santa Lucía (Cartagena):** Onofre Juan Martínez, Francisco Jesús Vera, Lorena Martínez, Josefina García, Begoña Alcaraz, Amaya Jimeno.

**Complejo Hospitalario Universitario a Coruña (Chuac) (A Coruña):** Angeles Castro Iglesias, Berta Pernas Souto, Alvaro Mena de Cea**.**

**Hospital Universitario Virgen de la Arrixaca (El Palmar):** Carlos Galera, Helena Albendin, Aurora Pérez, Asunción Iborra, Antonio Moreno, Maria Angustias Merlos, Asunción Vidal.

**Hospital Universitario Infanta Sofia (San Sebastian de los Reyes):** Inés Suárez-García, Eduardo Malmierca, Patricia González-Ruano, Dolores Martín Rodrigo, Mª Pilar Ruiz Seco.

**Complejo Hospitalario de Jaén (Jaén)** Mohamed Omar Mohamed-Balghata, María Amparo Gómez Vidal.

**Hospital Clínico San Carlos (Madrid):** Vicente Estrada Pérez, Maria Jesus Téllez Molina, Jorge Vergas García, Juncal Pérez-Somarriba Moreno

**Hospital Universitario Fundación Jiménez Díaz (Madrid):** Miguel Górgolas., Alfonso Cabello., Beatriz Álvarez., Laura Prieto.

**Hospital Universitario Príncipe de Asturias (Alcalá de Henares):** José Sanz Moreno, Alberto Arranz Caso, Cristina Hernández Gutiérrez, María Novella Mena.

**Hospital Clínico Universitario de Valencia (València):** María José Galindo Puerto, Ramón Fernando Vilalta, Ana Ferrer Ribera.

**Hospital Reina Sofía (Córdoba):** Antonio Rivero Román, Maria Teresa Brieva Herrero, Antonio Rivero Juárez, Pedro López López, Isabel Machuca Sánchez, José Peña Martínez.

**Hospital Universitario Severo Ochoa (Leganés)**: Miguel Cervero Jiménez, Rafael Torres Perea, Juan José Jusdado Ruiz-Capillas.

**Nuestra Señora de Valme:** Juan A Pineda.
