# Supplementary material for: DBP rs7041 and DHCR7 rs3829251 are Linked to CD4+ Recovery in HIV Patients on Antiretroviral Therapy
Source: Front Pharmacol. 2022 Jan 18;12:773848. doi: 10.3389/fphar.2021.773848 (PMC8804497; doi:10.3389/fphar.2021.773848)

**Supplemental Table 1.** Distribution of single nucleotide polymorphisms related to vitamin D pathway in HIV infected patients and healthy donors.

|  |  |  | **HWE** | |  | **HWE** | | **HIV vs. Control** | |
| --- | --- | --- | --- | --- | --- | --- | --- | --- | --- |
| **SNPs** | **Genotype** | **Control-group** | **p-value** | **q-value** | **HIV-group** | **p-value** | **q-value** | **p-value** | **q-value** |
| ***VDR* rs11568820** | *N/A* | 1 (0.7%) |  |  | 0 (0%) |  |  |  |  |
|  | C/C | 85 (58.6%) | 0.490 | 0.694 | 243 (59.1%) | 0.500 | 0.765 | 0.394 | 0.558 |
|  | C/T | 49 (33.8%) |  |  | 143 (34.8%) |  |  |  |  |
|  | T/T | 10 (6.9%) |  |  | 25 (6.1%) |  |  |  |  |
| ***VDR* rs1544410** | *N/A* | 1 (0.7%) |  |  | 0 (0%) |  |  |  |  |
|  | C/C | 49 (33.8%) | 0.310 | 0.586 | 161 (39.2%) | 0.520 | 0.765 | **0.035** | 0.293 |
|  | C/T | 65 (44.8%) |  |  | 198 (48.2%) |  |  |  |  |
|  | T/T | 30 (20.7%) |  |  | 52 (12.7%) |  |  |  |  |
| ***VDR* rs2228570** | *N/A* | 1 (0.7%) |  |  | 0 (0%) |  |  |  |  |
|  | A/A | 18 (12.4%) | 0.120 | 0.404 | 64 (15.6%) | 0.190 | 0.765 | 0.122 | 0.301 |
|  | A/G | 54 (37.2%) |  |  | 175 (42.6%) |  |  |  |  |
|  | G/G | 72 (49.7%) |  |  | 172 (41.8%) |  |  |  |  |
| ***VDR* rs4516035** | *N/A* | 0 (0%) |  |  | 2 (0.5%) |  |  |  |  |
|  | C/C | 29 (20%) | **0.037** | 0.404 | 66 (16.1%) | 0.410 | 0.765 | 0.437 | 0.571 |
|  | C/T | 57 (39.3%) |  |  | 186 (45.3%) |  |  |  |  |
|  | T/T | 59 (40.7%) |  |  | 157 (38.2%) |  |  |  |  |
| ***VDR* rs2238136** | *N/A* | 2 (1.4%) |  |  | 0 (0%) |  |  |  |  |
|  | C/C | 76 (52.4%) | 0.190 | 0.404 | 234 (56.9%) | 0.790 | 0.895 | 0.069 | 0.293 |
|  | C/T | 61 (42.1%) |  |  | 154 (37.5%) |  |  |  |  |
|  | T/T | 6 (4.1%) |  |  | 23 (5.6%) |  |  |  |  |
| ***VDR* rs7970314** | *N/A* | 1 (0.7%) |  |  | 1 (0.2%) |  |  |  |  |
|  | A/A | 80 (55.2%) | 0.830 | 0.941 | 230 (56%) | 0.520 | 0.765 | 0.893 | 0.893 |
|  | A/G | 54 (37.2%) |  |  | 151 (36.7%) |  |  |  |  |
|  | G/G | 10 (6.9%) |  |  | 29 (7.1%) |  |  |  |  |
| ***DBP* rs12512631** | *N/A* | 2 (1.4%) |  |  | 1 (0.2%) |  |  |  |  |
|  | C/C | 22 (15.2%) | 0.099 | 0.404 | 61 (14.8%) | 0.540 | 0.765 | 0.053 | 0.293 |
|  | C/T | 56 (38.6%) |  |  | 203 (49.4%) |  |  |  |  |
|  | T/T | 65 (44.8%) |  |  | 146 (35.5%) |  |  |  |  |
| ***DBP* rs16846876** | *N/A* | 1 (0.7%) |  |  | 2 (0.5%) |  |  |  |  |
|  | A/A | 59 (40.7%) | 0.720 | 0.941 | 200 (48.7%) | 0.480 | 0.765 | 0.124 | 0.301 |
|  | A/T | 65 (44.8%) |  |  | 177 (43.1%) |  |  |  |  |
|  | T/T | 20 (13.8%) |  |  | 32 (7.8%) |  |  |  |  |
| ***DBP* rs7041** | *N/A* | 1 (0.7%) |  |  | 0 (0%) |  |  |  |  |
|  | A/A | 37 (25.5%) | 0.410 | 0.634 | 81 (19.7%) | 0.230 | 0.765 | 0.142 | 0.302 |
|  | A/C | 67 (46.2%) |  |  | 217 (52.8%) |  |  |  |  |
|  | C/C | 40 (27.6%) |  |  | 113 (27.5%) |  |  |  |  |
| ***CYP2R1* rs1993116** | *N/A* | 1 (0.7%) |  |  | 1 (0.2%) |  |  |  |  |
|  | A/A | 29 (20%) | 0.120 | 0.404 | 64 (15.6%) | 0.350 | 0.765 | 0.534 | 0.648 |
|  | A/G | 60 (41.4%) |  |  | 184 (44.8%) |  |  |  |  |
|  | G/G | 55 (37.9%) |  |  | 162 (39.4%) |  |  |  |  |
| ***CYP27A1* rs17470271** | *N/A* | 2 (1.4%) |  |  | 1 (0.2%) |  |  |  |  |
|  | A/A | 48 (33.1%) | 0.065 | 0.404 | 134 (32.6%) | 0.690 | 0.838 | 0.185 | 0.349 |
|  | A/T | 60 (41.4%) |  |  | 197 (47.9%) |  |  |  |  |
|  | T/T | 35 (24.1%) |  |  | 79 (19.2%) |  |  |  |  |
| ***CYP27B1* rs10877012** | *N/A* | 1 (0.7%) |  |  | 0 (0%) |  |  |  |  |
|  | G/G | 85 (58.6%) | 0.180 | 0.404 | 233 (56.7%) | 0.430 | 0.765 | 0.312 | 0.530 |
|  | G/T | 47 (32.4%) |  |  | 149 (36.3%) |  |  |  |  |
|  | T/T | 12 (8.3%) |  |  | 29 (7.1%) |  |  |  |  |
| ***CYP24A1* rs6013897** | A/A | 2 (1.4%) | 0.160 | 0.404 | 32 (7.8%) | 0.062 | 0.527 | **0.019** |  |
|  | A/T | 49 (33.8%) |  |  | 138 (33.6%) |  |  |  | 0.293 |
|  | T/T | 94 (64.8%) |  |  | 241 (58.6%) |  |  |  |  |
| ***CYP3A4* rs2740574** | *N/A* | 2 (1.4%) |  |  | 0 (0%) |  |  |  |  |
|  | C/C | 1 (0.7%) | 0.360 | 0.612 | 4 (1%) | **0.046** | 0.527 | 0.123 | 0.301 |
|  | C/T | 14 (9.7%) |  |  | 41 (10%) |  |  |  |  |
|  | T/T | 128 (88.3%) |  |  | 366 (89.1%) |  |  |  |  |
| ***RXRA* rs7861779** | *N/A* | 2 (1.4%) |  |  | 5 (1.2%) |  |  |  | 0.722 |
|  | C/C | 103 (71%) | 0.999 | 0.999 | 314 (76.4%) | 0.999 | 0.999 | 0.637 |  |
|  | C/T | 37 (25.5%) |  |  | 86 (20.9%) |  |  |  |  |
|  | T/T | 3 (2.1%) |  |  | 6 (1.5%) |  |  |  |  |
| ***DHCR7* rs12785878** | *N/A* | 1 (0.7%) |  |  | 0 (0%) |  |  |  |  |
|  | A/A | 18 (12.4%) | 0.999 | 0.999 | 57 (13.9%) | 0.670 | 0.838 | 0.390 | 0.558 |
|  | A/G | 66 (45.5%) |  |  | 186 (45.2%) |  |  |  |  |
|  | G/G | 60 (41.4%) |  |  | 168 (40.9%) |  |  |  |  |
| ***DHCR7* rs3829251** | A/A | 6 (4.1%) | 0.790 | 0.941 | 16 (3.9%) | 0.880 | 0.935 | 0.856 | 0.893 |
|  | A/G | 44 (30.3%) |  |  | 135 (32.8%) |  |  |  |  |
|  | G/G | 95 (65.5%) |  |  | 260 (63.3%) |  |  |  |  |

**Statistics**: P-values were calculated by the Chi-squared test. Significant values are shown in bold.

**Abbreviations**: HIV; human immunodeficiency virus; HWE, Hardy-Weinberg equilibrium; VDR, vitamin D receptor; DBP, vitamin D-binding protein; CYP2R1, cytochrome P450 family 2 subfamily R member 1; CYP27A1, cytochrome P450 family 27 subfamily A member 1; CYP27B1, cytochrome P450 25-hydroxyvitamin D_3_ 1 alpha-hydroxylase; CYP24A1, cytochrome P450 family 24 subfamily A member 1; CYP3A4, cytochrome P450 Family 3 Subfamily A member 4; RXRA, retinoid X receptor-alpha; DHCR7, 7-Dehidrocolesterol reductase; p-value, level of significance; q-values, p-values corrected for multiple testing using the false discovery rate (FDR) with Benjamini and Hochberg procedure; N/A, not available.

**Supplemental Table 2.** Summary of univariate regressions between vitamin D pathway single nucleotide polymorphisms (SNPs) and CD4^+^ T-cells recovery in HIV-infected patients who started ART with very low CD4+T-cells count (<200 cells/mm^3^).

|  |  |  | **Dominant** | | | | **Recessive** | | | | **Additive** | | | |
| --- | --- | --- | --- | --- | --- | --- | --- | --- | --- | --- | --- | --- | --- | --- |
|  | **Gene** | **SNP** | **Exp(b)** | **95%CI** | **p** | **q** | **Exp(b)** | **95%CI** | **p** | **q** | **Exp(b)** | **95%CI** | **p** | **q** |
| **CD4^+^ T-cell count increased** | VDR | rs11568820 | 0.90 | (0.79; 1.02) | 0.095 | 0.615 | 0.89 | (0.69; 1.16) | 0.393 | 0.601 | 0.92 | (0.83; 1.01) | 0.091 | 0.317 |
|  | VDR | rs1544410 | 1.08 | (0.95; 1.22) | 0.244 | 0.742 | 1.21 | (1.01; 1.46) | ***0.037*** | 0.210 | 1.09 | (1.00; 1.19) | 0.057 | 0.317 |
|  | VDR | rs2228570 | 1.09 | (0.96; 1.24) | 0.172 | 0.732 | 1.13 | (0.96; 1.34) | 0.153 | 0.587 | 1.08 | (0.99; 1.17) | 0.093 | 0.317 |
|  | VDR | rs4516035 | 1.13 | (1.00; 1.28) | 0.059 | 0.615 | 1.10 | (0.93; 1.30) | 0.279 | 0.601 | 1.09 | (1.00; 1.19) | 0.062 | 0.317 |
|  | VDR | rs2238136 | 1.11 | (0.98; 1.25) | 0.108 | 0.615 | 1.07 | (0.82; 1.40) | 0.611 | 0.800 | 1.08 | (0.98; 1.20) | 0.130 | 0.368 |
|  | VDR | rs7970314 | 0.94 | (0.83; 1.06) | 0.307 | 0.742 | 0.91 | (0.71; 1.15) | 0.424 | 0.601 | 0.94 | (0.86; 1.04) | 0.255 | 0.541 |
|  | DBP | rs12512631 | 1.01 | (0.89; 1.15) | 0.897 | 0.948 | 1.03 | (0.87; 1.23) | 0.708 | 0.859 | 1.01 | (0.93; 1.11) | 0.773 | 0.884 |
|  | DBP | rs16846876 | 1.06 | (0.94; 1.20) | 0.349 | 0.742 | 0.99 | (0.78; 1.24) | 0.904 | 0.904 | 1.04 | (0.94; 1.14) | 0.489 | 0.727 |
|  | DBP | rs7041 | 1.04 | (0.90; 1.19) | 0.615 | 0.948 | 1.25 | (1.08; 1.46) | ***0.004*** | ***0.040*** | 1.10 | (1.01; 1.19) | ***0.039*** | 0.317 |
|  | CYP2R1 | rs1993116 | 0.98 | (0.87; 1.12) | 0.806 | 0.948 | 1.08 | (0.91; 1.28) | 0.392 | 0.601 | 1.01 | (0.93; 1.10) | 0.780 | 0.884 |
|  | CYP27A1 | rs17470271 | 0.97 | (0.85; 1.11) | 0.636 | 0.948 | 0.91 | (0.78; 1.06) | 0.235 | 0.601 | 0.96 | (0.88; 1.05) | 0.331 | 0.563 |
|  | CYP27B1 | rs10877012 | 1.00 | (0.88; 1.13) | 0.944 | 0.948 | 1.18 | (0.93; 1.50) | 0.173 | 0.587 | 1.03 | (0.93; 1.13) | 0.601 | 0.786 |
|  | CYP24A1 | rs6013897 | 1.07 | (0.94; 1.21) | 0.305 | 0.742 | 1.11 | (0.88; 1.40) | 0.361 | 0.601 | 1.06 | (0.96; 1.17) | 0.240 | 0.541 |
|  | CYP3A4 | rs2740574 | 1.01 | (0.83; 1.23) | 0.942 | 0.948 | 0.74 | (0.40; 1.39) | 0.350 | 0.601 | 0.99 | (0.83; 1.18) | 0.869 | 0.916 |
|  | RXRA | rs7861779 | 0.94 | (0.81; 1.09) | 0.424 | 0.800 | 1.07 | (0.64; 1.79) | 0.808 | 0.904 | 0.96 | (0.84; 1.09) | 0.514 | 0.727 |
|  | DHCR7 | rs12785878 | 0.99 | (0.87; 1.12) | 0.815 | 0.948 | 1.01 | (0.85; 1.21) | 0.903 | 0.904 | 1.00 | (0.91; 1.09) | 0.916 | 0.916 |
|  | DHCR7 | rs3829251 | 1.00 | (0.88; 1.13) | 0.948 | 0.948 | 1.57 | (1.15; 2.15) | ***0.005*** | ***0.040*** | 1.06 | (0.95; 1.17) | 0.293 | 0.553 |
|  | **Gene** | **SNP** | **Exp(b)** | **95%CI** | **p** | **q** | **Exp(b)** | **95%CI** | **p** | **q** | **Exp(b)** | **95%CI** | **p** | **q** |
| **CD4^+^ T-cell count increased ≥P75th** | VDR | rs11568820 | 0.62 | (0.39; 0.99) | ***0.044*** | 0.185 | 0.40 | (0.12; 1.35) | 0.138 | 0.386 | 0.64 | (0.43; 0.95) | ***0.027*** | 0.172 |
|  | VDR | rs1544410 | 1.11 | (0.70; 1.77) | 0.647 | 0.843 | 1.57 | (0.84; 2.94) | 0.162 | 0.386 | 1.19 | (0.85; 1.66) | 0.302 | 0.428 |
|  | VDR | rs2228570 | 1.53 | (0.96; 2.43) | 0.076 | 0.185 | 1.12 | (0.61; 2.04) | 0.725 | 0.862 | 1.25 | (0.92; 1.71) | 0.158 | 0.245 |
|  | VDR | rs4516035 | 1.73 | (1.06; 2.82) | ***0.027*** | 0.185 | 1.56 | (0.88; 2.77) | 0.130 | 0.386 | 1.46 | (1.06; 2.00) | ***0.021*** | 0.172 |
|  | VDR | rs2238136 | 1.70 | (1.08; 2.67) | ***0.021*** | 0.185 | 1.07 | (0.41; 2.80) | 0.885 | 0.944 | 1.44 | (1.00; 2.06) | 0.050 | 0.172 |
|  | VDR | rs7970314 | 0.64 | (0.40; 1.01) | 0.055 | 0.185 | 0.47 | (0.16; 1.38) | 0.169 | 0.386 | 0.66 | (0.45; 0.98) | ***0.037*** | 0.172 |
|  | DBP | rs12512631 | 0.84 | (0.53; 1.34) | 0.468 | 0.663 | 1.10 | (0.59; 2.05) | 0.754 | 0.862 | 0.94 | (0.68; 1.31) | 0.728 | 0.860 |
|  | DBP | rs16846876 | 1.02 | (0.65; 1.60) | 0.929 | 0.968 | 1.02 | (0.44; 2.34) | 0.967 | 0.967 | 1.02 | (0.71; 1.45) | 0.930 | 0.930 |
|  | DBP | rs7041 | 0.94 | (0.57; 1.55) | 0.807 | 0.914 | 2.43 | (1.45; 4.07) | ***0.001*** | ***0.013*** | 1.36 | (0.98; 1.89) | 0.068 | 0.192 |
|  | CYP2R1 | rs1993116 | 1.34 | (0.84; 2.14) | 0.216 | 0.408 | 1.60 | (0.90; 2.85) | 0.112 | 0.386 | 1.31 | (0.96; 1.80) | 0.093 | 0.192 |
|  | CYP27A1 | rs17470271 | 1.28 | (0.78; 2.09) | 0.328 | 0.557 | 0.81 | (0.45; 1.46) | 0.475 | 0.760 | 1.04 | (0.76; 1.43) | 0.802 | 0.860 |
|  | CYP27B1 | rs10877012 | 0.99 | (0.63; 1.56) | 0.968 | 0.968 | 1.40 | (0.62; 3.18) | 0.423 | 0.752 | 1.06 | (0.74; 1.51) | 0.766 | 0.860 |
|  | CYP24A1 | rs6013897 | 1.51 | (0.97; 2.38) | 0.071 | 0.185 | 1.20 | (0.54; 2.69) | 0.652 | 0.862 | 1.32 | (0.94; 1.85) | 0.113 | 0.192 |
|  | CYP3A4 | rs2740574 | 0.53 | (0.23; 1.22) | 0.133 | 0.283 | *N/A* | *N/A* | *N/A* | *N/A* | 0.52 | (0.23; 1.14) | 0.102 | 0.192 |
|  | RXRA | rs7861779 | 0.53 | (0.29; 0.98) | ***0.042*** | 0.185 | 0.62 | (0.07; 5.34) | 0.660 | 0.862 | 0.57 | (0.32; 1.00) | 0.051 | 0.172 |
|  | DHCR7 | rs12785878 | 1.10 | (0.69; 1.73) | 0.694 | 0.843 | 0.69 | (0.34; 1.39) | 0.301 | 0.601 | 0.96 | (0.69; 1.33) | 0.810 | 0.860 |
|  | DHCR7 | rs3829251 | 1.22 | (0.77; 1.93) | 0.404 | 0.624 | 4.18 | (1.51; 11.52) | ***0.006*** | ***0.046*** | 1.40 | (0.95; 2.05) | 0.085 | 0.192 |
|  | **Gene** | **SNP** | **Exp(b)** | **95%CI** | **p** | **q** | **Exp(b)** | **95%CI** | **p** | **q** | **Exp(b)** | **95%CI** | **p** | **q** |
| **Slope of CD4^+^ T-cells recovery** | VDR | rs11568820 | 0.93 | (0.81; 1.06) | 0.244 | 0.779 | 1.01 | (0.77; 1.32) | 0.942 | 0.942 | 0.95 | (0.86; 1.06) | 0.365 | 0.759 |
|  | VDR | rs1544410 | 1.11 | (0.97; 1.26) | 0.121 | 0.688 | 1.21 | (1.00; 1.46) | 0.054 | 0.231 | 1.11 | (1.01; 1.22) | ***0.036*** | 0.151 |
|  | VDR | rs2228570 | 1.13 | (0.99; 1.29) | 0.067 | 0.568 | 1.19 | (1.00; 1.42) | ***0.050*** | 0.231 | 1.11 | (1.02; 1.21) | ***0.023*** | 0.141 |
|  | VDR | rs4516035 | 1.18 | (1.04; 1.35) | ***0.012*** | 0.207 | 1.10 | (0.92; 1.31) | 0.310 | 0.586 | 1.11 | (1.01; 1.22) | ***0.025*** | 0.141 |
|  | VDR | rs2238136 | 1.06 | (0.93; 1.20) | 0.413 | 0.779 | 1.03 | (0.78; 1.36) | 0.842 | 0.942 | 1.04 | (0.94; 1.16) | 0.453 | 0.759 |
|  | VDR | rs7970314 | 0.96 | (0.84; 1.09) | 0.529 | 0.824 | 1.02 | (0.80; 1.32) | 0.863 | 0.942 | 0.98 | (0.88; 1.08) | 0.670 | 0.759 |
|  | DBP | rs12512631 | 1.02 | (0.89; 1.17) | 0.783 | 0.832 | 1.02 | (0.85; 1.22) | 0.853 | 0.942 | 1.01 | (0.92; 1.12) | 0.771 | 0.816 |
|  | DBP | rs16846876 | 1.06 | (0.94; 1.21) | 0.345 | 0.779 | 0.91 | (0.71; 1.15) | 0.426 | 0.725 | 1.02 | (0.92; 1.14) | 0.666 | 0.759 |
|  | DBP | rs7041 | 1.07 | (0.93; 1.24) | 0.334 | 0.779 | 1.30 | (1.11; 1.52) | ***0.001*** | ***0.021*** | 1.12 | (1.03; 1.23) | ***0.011*** | 0.141 |
|  | CYP2R1 | rs1993116 | 0.98 | (0.86; 1.11) | 0.720 | 0.832 | 1.15 | (0.96; 1.37) | 0.123 | 0.349 | 1.03 | (0.94; 1.12) | 0.569 | 0.759 |
|  | CYP27A1 | rs17470271 | 0.98 | (0.85; 1.12) | 0.770 | 0.832 | 0.91 | (0.77; 1.07) | 0.245 | 0.521 | 0.96 | (0.88; 1.05) | 0.403 | 0.759 |
|  | CYP27B1 | rs10877012 | 1.03 | (0.90; 1.17) | 0.682 | 0.832 | 1.26 | (0.98; 1.62) | 0.068 | 0.231 | 1.06 | (0.96; 1.17) | 0.271 | 0.658 |
|  | CYP24A1 | rs6013897 | 1.08 | (0.95; 1.23) | 0.264 | 0.779 | 1.18 | (0.93; 1.50) | 0.173 | 0.420 | 1.08 | (0.97; 1.19) | 0.150 | 0.509 |
|  | CYP3A4 | rs2740574 | 1.10 | (0.90; 1.35) | 0.366 | 0.779 | 0.83 | (0.43; 1.60) | 0.582 | 0.899 | 1.07 | (0.89; 1.28) | 0.496 | 0.759 |
|  | RXRA | rs7861779 | 0.95 | (0.81; 1.11) | 0.533 | 0.824 | 1.07 | (0.62; 1.84) | 0.801 | 0.942 | 0.97 | (0.84; 1.11) | 0.623 | 0.759 |
|  | DHCR7 | rs12785878 | 1.03 | (0.90; 1.17) | 0.691 | 0.832 | 0.99 | (0.82; 1.19) | 0.918 | 0.942 | 1.01 | (0.92; 1.11) | 0.816 | 0.816 |
|  | DHCR7 | rs3829251 | 1.01 | (0.89; 1.16) | 0.863 | 0.863 | 1.59 | (1.15; 2.21) | ***0.005*** | ***0.047*** | 1.07 | (0.96; 1.19) | 0.219 | 0.622 |
|  | **Gene** | **SNP** | **Exp(b)** | **95%CI** | **p** | **q** | **Exp(b)** | **95%CI** | **p** | **q** | **Exp(b)** | **95%CI** | **p** | **q** |
| **Slope of CD4^+^ T-cells recovery ≥P75th** | VDR | rs11568820 | 0.69 | (0.43; 1.10) | 0.121 | 0.517 | 1.19 | (0.48; 2.94) | 0.704 | 0.773 | 0.81 | (0.55; 1.18) | 0.270 | 0.578 |
|  | VDR | rs1544410 | 1.11 | (0.70; 1.77) | 0.647 | 0.912 | 2.11 | (1.15; 3.89) | ***0.016*** | 0.088 | 1.30 | (0.93; 1.81) | 0.124 | 0.435 |
|  | VDR | rs2228570 | 1.16 | (0.73; 1.83) | 0.534 | 0.826 | 1.12 | (0.61; 2.04) | 0.725 | 0.773 | 1.10 | (0.81; 1.51) | 0.541 | 0.708 |
|  | VDR | rs4516035 | 1.56 | (0.97; 2.53) | 0.068 | 0.517 | 1.29 | (0.72; 2.32) | 0.401 | 0.761 | 1.32 | (0.96; 1.81) | 0.089 | 0.435 |
|  | VDR | rs2238136 | 1.18 | (0.75; 1.85) | 0.479 | 0.814 | 1.35 | (0.54; 3.38) | 0.522 | 0.764 | 1.17 | (0.81; 1.68) | 0.408 | 0.578 |
|  | VDR | rs7970314 | 0.69 | (0.44; 1.10) | 0.120 | 0.517 | 1.39 | (0.61; 3.17) | 0.428 | 0.761 | 0.84 | (0.58; 1.22) | 0.362 | 0.578 |
|  | DBP | rs12512631 | 0.94 | (0.59; 1.50) | 0.805 | 0.912 | 1.22 | (0.66; 2.25) | 0.526 | 0.764 | 1.03 | (0.74; 1.43) | 0.874 | 0.926 |
|  | DBP | rs16846876 | 1.08 | (0.69; 1.69) | 0.750 | 0.912 | 0.69 | (0.27; 1.72) | 0.419 | 0.761 | 0.98 | (0.69; 1.40) | 0.926 | 0.926 |
|  | DBP | rs7041 | 1.00 | (0.61; 1.66) | 0.991 | 0.991 | 2.60 | (1.55; 4.36) | ***0.000*** | ***0.005*** | 1.44 | (1.03; 2.00) | ***0.031*** | 0.290 |
|  | CYP2R1 | rs1993116 | 1.07 | (0.68; 1.70) | 0.761 | 0.912 | 1.46 | (0.82; 2.62) | 0.201 | 0.689 | 1.15 | (0.84; 1.58) | 0.382 | 0.578 |
|  | CYP27A1 | rs17470271 | 1.22 | (0.75; 1.99) | 0.417 | 0.787 | 0.87 | (0.49; 1.55) | 0.632 | 0.773 | 1.05 | (0.76; 1.43) | 0.786 | 0.891 |
|  | CYP27B1 | rs10877012 | 0.99 | (0.63; 1.56) | 0.968 | 0.991 | 1.66 | (0.75; 3.70) | 0.215 | 0.689 | 1.09 | (0.77; 1.55) | 0.631 | 0.767 |
|  | CYP24A1 | rs6013897 | 1.23 | (0.78; 1.93) | 0.377 | 0.787 | 1.42 | (0.65; 3.10) | 0.382 | 0.761 | 1.20 | (0.85; 1.70) | 0.294 | 0.578 |
|  | CYP3A4 | rs2740574 | 1.43 | (0.73; 2.80) | 0.302 | 0.734 | *N/A* | *N/A* | *N/A* | *N/A* | 1.32 | (0.73; 2.40) | 0.360 | 0.578 |
|  | RXRA | rs7861779 | 0.63 | (0.35; 1.13) | 0.122 | 0.517 | 0.61 | (0.07; 5.27) | 0.652 | 0.773 | 0.66 | (0.38; 1.13) | 0.128 | 0.435 |
|  | DHCR7 | rs12785878 | 1.29 | (0.81; 2.05) | 0.276 | 0.734 | 1.10 | (0.58; 2.07) | 0.778 | 0.778 | 1.16 | (0.84; 1.61) | 0.358 | 0.578 |
|  | DHCR7 | rs3829251 | 1.36 | (0.86; 2.14) | 0.191 | 0.651 | 4.18 | (1.51; 11.52) | ***0.006*** | ***0.046*** | 1.51 | (1.03; 2.21) | ***0.034*** | 0.290 |
|  | **Gene** | **SNP** | **Exp(b)** | **95%CI** | **p** | **q** | **Exp(b)** | **95%CI** | **p** | **q** | **Exp(b)** | **95%CI** | **p** | **q** |
| **Achieve ≥500 CD4^+^T-cells/mm^3^** | VDR | rs11568820 | 0.72 | (0.46; 1.13) | 0.151 | 0.366 | 0.66 | (0.24; 1.80) | 0.418 | 0.642 | 0.75 | (0.52; 1.10) | 0.140 | 0.297 |
|  | VDR | rs1544410 | 1.41 | (0.89; 2.23) | 0.141 | 0.366 | 1.67 | (0.91; 3.08) | 0.100 | 0.321 | 1.37 | (0.99; 1.89) | 0.059 | 0.199 |
|  | VDR | rs2228570 | 1.55 | (0.99; 2.44) | 0.058 | 0.308 | 1.40 | (0.79; 2.48) | 0.256 | 0.504 | 1.34 | (0.99; 1.82) | 0.058 | 0.199 |
|  | VDR | rs4516035 | 1.53 | (0.96; 2.44) | 0.072 | 0.308 | 1.73 | (0.99; 3.03) | 0.053 | 0.284 | 1.43 | (1.05; 1.95) | ***0.024*** | 0.199 |
|  | VDR | rs2238136 | 1.30 | (0.84; 2.01) | 0.244 | 0.376 | 0.74 | (0.27; 2.04) | 0.559 | 0.696 | 1.14 | (0.80; 1.64) | 0.462 | 0.620 |
|  | VDR | rs7970314 | 0.76 | (0.49; 1.19) | 0.235 | 0.376 | 0.70 | (0.28; 1.76) | 0.441 | 0.642 | 0.79 | (0.55; 1.14) | 0.209 | 0.355 |
|  | DBP | rs12512631 | 0.96 | (0.61; 1.51) | 0.847 | 0.887 | 1.17 | (0.64; 2.13) | 0.609 | 0.696 | 1.02 | (0.74; 1.41) | 0.895 | 0.895 |
|  | DBP | rs16846876 | 1.09 | (0.71; 1.69) | 0.690 | 0.838 | 0.61 | (0.24; 1.51) | 0.283 | 0.504 | 0.98 | (0.69; 1.38) | 0.885 | 0.895 |
|  | DBP | rs7041 | 0.76 | (0.47; 1.23) | 0.266 | 0.376 | 2.08 | (1.24; 3.46) | ***0.005*** | ***0.042*** | 1.16 | (0.84; 1.60) | 0.359 | 0.555 |
|  | CYP2R1 | rs1993116 | 0.94 | (0.61; 1.47) | 0.795 | 0.887 | 1.65 | (0.94; 2.89) | 0.084 | 0.321 | 1.12 | (0.82; 1.52) | 0.474 | 0.620 |
|  | CYP27A1 | rs17470271 | 1.33 | (0.83; 2.15) | 0.240 | 0.376 | 0.91 | (0.52; 1.59) | 0.736 | 0.785 | 1.10 | (0.81; 1.49) | 0.555 | 0.628 |
|  | CYP27B1 | rs10877012 | 1.35 | (0.87; 2.08) | 0.185 | 0.376 | 1.72 | (0.79; 3.78) | 0.174 | 0.463 | 1.32 | (0.94; 1.86) | 0.107 | 0.296 |
|  | CYP24A1 | rs6013897 | 1.43 | (0.92; 2.22) | 0.111 | 0.366 | 1.06 | (0.48; 2.37) | 0.882 | 0.882 | 1.25 | (0.89; 1.74) | 0.195 | 0.355 |
|  | CYP3A4 | rs2740574 | 0.46 | (0.20; 1.07) | 0.072 | 0.308 | *N/A* | *N/A* | *N/A* | *N/A* | 0.46 | (0.21; 1.02) | 0.056 | 0.199 |
|  | RXRA | rs7861779 | 0.56 | (0.31; 0.99) | ***0.047*** | 0.308 | 0.55 | (0.06; 4.74) | 0.585 | 0.696 | 0.59 | (0.34; 1.00) | 0.052 | 0.199 |
|  | DHCR7 | rs12785878 | 0.97 | (0.62; 1.51) | 0.887 | 0.887 | 0.69 | (0.35; 1.35) | 0.277 | 0.504 | 0.90 | (0.65; 1.24) | 0.517 | 0.627 |
|  | DHCR7 | rs3829251 | 1.12 | (0.72; 1.76) | 0.609 | 0.797 | 4.85 | (1.72; 13.69) | ***0.003*** | ***0.042*** | 1.34 | (0.92; 1.96) | 0.122 | 0.296 |

**Statistical**: Values were calculated by univariate regressions (GLM models, see statistical analysis section). Significant values are shown in bold.

**Abbreviations**: Exp(b), exponentiation of the beta coefficient, which was arithmetic mean ratio (AMR) for continuous variables and odds ratio (OR) for categorical variables; VDR, vitamin D receptor; DBP, vitamin D-binding protein; CYP2R1, cytochrome P450 family 2 subfamily R member 1; CYP27A1, cytochrome P450 family 27 subfamily A member 1; CYP27B1, cytochrome P450 25-hydroxyvitamin D_3_ 1 alpha-hydroxylase; CYP24A1, cytochrome P450 family 24 subfamily A member 1; CYP3A4, cytochrome P450 Family 3 Subfamily A member 4; RXRA, retinoid X receptor-alpha; DHCR7, 7-Dehidrocolesterol reductase; HIV, human immunodeficiency virus; 95%CI, 95% of confidence interval; p-value, level of significance; q-values, p-values corrected for multiple testing using the false discovery rate (FDR) with Benjamini and Hochberg procedure; N/A, not available.

**Supplemental Table 3.** Association of *VDR*, *DBP,* and *DHCR7* haplotypes with CD4^+^ T-cells recovery in HIV-infected patients who started ART with very low CD4+T-cells count (<200 cells/mm^3^).

|  |  | **CD4^+^ T-cell count increased ≥P75th** | | | **Slope of CD4^+^ T-cells recovery ≥P75th** | | | **Achieve ≥500 CD4^+^T-cells/mm^3^** | | |
| --- | --- | --- | --- | --- | --- | --- | --- | --- | --- | --- |
| ***VDR* Haplotype** | **Freq.** | **OR (95% CI)** | ***p*-value** | ***q*-value** | **OR (95% CI)** | ***p*-value** | ***q*-value** | **OR (95% CI)** | ***p*-value** | ***q*-value** |
| CGTTTG | 0.015 | 0.66 (0.11; 3.80) | 0.629 | 0.815 | 0.83 (0.16; 4.36) | 0.822 | 0.993 | 0.59 (0.10; 3.36) | 0.534 | 0.860 |
| TACTTG | 0.041 | 0.56 (0.18; 1.75) | 0.298 | 0.815 | 0.99 (0.35; 2.80) | 0.993 | 0.995 | 0.86 (0.31; 2.40) | 0.772 | 0.896 |
| CACTTG | 0.043 | 0.97 (0.33; 2.91) | 0.963 | 0.963 | 0.76 (0.24; 2.38) | 0.633 | 0.993 | 1.25 (0.45; 3.50) | 0.674 | 0.874 |
| TGCTTG | 0.053 | 0.57 (0.21; 1.53) | 0.239 | 0.815 | **0.35 (0.11; 1.09)** | **0.044** | 0.319 | 0.86 (0.36; 2.02) | 0.723 | 0.874 |
| CGCTTG | 0.077 | **0.32 (0.12; 0.83)** | **0.010** | 0.815 | 0.91 (0.43; 1.90) | 0.800 | 0.993 | **0.34 (0.13; 0.84)** | **0.010** | 0.135 |
| CACTCG | 0.010 | 0.59 (0.08; 4.35) | 0.584 | 0.815 | 0.57 (0.08; 4.31) | 0.570 | 0.993 | 0.97 (0.17; 5.49) | 0.976 | 0.984 |
| CATCCA | 0.018 | 2.65 (0.60; 11.6) | 0.209 | 0.815 | 1.04 (0.20; 5.36) | 0.962 | 0.995 | 1.55 (0.34; 7.08) | 0.577 | 0.874 |
| CGTCCA | 0.018 | 3.89 (0.89; 16.90) | 0.076 | 0.815 | 2.60 (0.59; 11.50) | 0.220 | 0.638 | 3.13 (0.72; 13.5) | 0.133 | 0.764 |
| TACCCA | 0.049 | **2.95 (1.24; 7.02)** | **0.015** | 0.815 | **2.52 (1.06; 5.99)** | **0.038** | 0.319 | **3.63 (1.52; 8.65)** | **0.003** | 0.087 |
| CACCCA | 0.067 | 1.37 (0.66; 2.82) | 0.407 | 0.815 | 1.00 (0.46; 2.17) | 0.995 | 0.995 | 1.54 (0.76; 3.10) | 0.237 | 0.764 |
| TGCCCA | 0.091 | 1.43 (0.75; 2.70) | 0.283 | 0.815 | 1.75 (0.93; 3.27) | 0.087 | 0.365 | 1.48 (0.80; 2.77) | 0.220 | 0.764 |
| CGCCCA | 0.134 | 0.80 (0.46; 1.40) | 0.431 | 0.815 | 0.75 (0.42; 1.32) | 0.306 | 0.740 | 0.72 (0.41; 1.26) | 0.236 | 0.764 |
| TATTCA | 0.014 | 1.82 (0.25; 13.5) | 0.564 | 0.815 | 2.19 (0.30; 15.7) | 0.447 | 0.926 | 2.23 (0.32; 15.5) | 0.426 | 0.860 |
| CATTCA | 0.059 | 1.75 (0.79; 3.87) | 0.172 | 0.815 | 0.94 (0.40; 2.22) | 0.894 | 0.995 | 1.18 (0.53; 2.64) | 0.690 | 0.874 |
| TGTTCA | 0.040 | 0.84 (0.31; 2.24) | 0.724 | 0.852 | 1.03 (0.41; 2.61) | 0.948 | 0.995 | 0.98 (0.39; 2.46) | 0.974 | 0.984 |
| CGTTCA | 0.062 | 1.29 (0.58; 2.88) | 0.535 | 0.815 | 0.89 (0.38; 2.09) | 0.789 | 0.993 | 0.67 (0.28; 1.61) | 0.353 | 0.860 |
| CACTCA | 0.047 | 0.54 (0.19; 1.49) | 0.200 | 0.815 | 0.80 (0.33; 1.94) | 0.611 | 0.993 | 0.73 (0.30; 1.77) | 0.472 | 0.860 |
| TGCTCA | 0.050 | 0.77 (0.29; 2.08) | 0.606 | 0.815 | 0.82 (0.31; 2.19) | 0.694 | 0.993 | 0.68 (0.25; 1.82) | 0.431 | 0.860 |
| CGCTCA | 0.078 | 0.62 (0.27; 1.40) | 0.232 | 0.815 | 0.60 (0.26; 1.37) | 0.213 | 0.638 | 0.65 (0.29; 1.42) | 0.267 | 0.774 |
| ***DBP* Haplotype** | **Freq.** | **OR (95% CI)** | ***p*-value** |  | **OR (95% CI)** | ***p*-value** |  | **OR (95% CI)** | ***p*-value** |  |
| TCA | 0.014 | 1.15 (0.29; 4.56) | 0.842 | 0.872 | 1.20 (0.31; 4.69) | 0.792 | 0.993 | 1.06 (0.27; 4.19) | 0.934 | 0.984 |
| ACA | 0.021 | 1.72 (0.59; 4.96) | 0.330 | 0.815 | **2.97 (1.03; 8.58)** | **0.044** | 0.319 | 1.48 (0.51; 4.28) | 0.481 | 0.860 |
| TTA | 0.245 | 1.15 (0.78; 1.68) | 0.482 | 0.815 | 1.11 (0.75; 1.62) | 0.605 | 0.993 | 1.07 (0.74; 1.56) | 0.714 | 0.874 |
| ATA | 0.182 | 1.28 (0.84; 1.93) | 0.250 | 0.815 | 1.33 (0.88; 2.01) | 0.177 | 0.638 | 1.11 (0.74; 1.67) | 0.624 | 0.874 |
| ACC | 0.357 | 0.90 (0.64; 1.27) | 0.555 | 0.815 | 0.93 (0.66; 1.31) | 0.678 | 0.993 | 1.00 (0.72; 1.40) | 0.984 | 0.984 |
| TTC | 0.032 | 0.36 (0.09; 1.43) | 0.106 | 0.815 | 0.34 (0.08; 1.37) | 0.088 | 0.365 | 0.49 (0.15; 1.62) | 0.210 | 0.764 |
| ATC | 0.145 | 0.78(0.47; 1.29) | 0.319 | 0.815 | 0.64 (0.38; 1.08) | 0.084 | 0.365 | 0.85 (0.53; 1.38) | 0.515 | 0.860 |
| ***DHCR7* Haplotype** | **Freq.** | **OR (95% CI)** | ***p*-value** |  | **OR (95% CI)** | ***p*-value** |  | **OR (95% CI)** | ***p*-value** |  |
| GA | 0.203 | 1.40 (0.95; 2.05) | 0.088 | 0.815 | **1.51 (1.03; 2.21)** | **0.036** | 0.319 | 1.34 (0.92; 1.95) | 0.125 | 0.764 |
| GG | 0.162 | **0.62 (0.39; 0.98)** | **0.034** | 0.815 | 0.80 (0.52; 1.22) | 0.297 | 0.740 | **0.58 (0.37; 0.92)** | **0.014** | 0.135 |
| TG | 0.635 | 1.04 (0.75; 1.44) | 0.810 | 0.870 | 0.86 (0.62; 1.19) | 0.359 | 0.801 | 1.11 (0.81; 1.53) | 0.516 | 0.860 |

**Statistics**: Values are expressed as odds ratio and 95% confidence interval. Associations were calculated by logistic regression. Only haplotypes with frequency >0.01 are shown. Significant differences are shown in bold. Haplotypes are composed by the following SNPs: i) *VDR* haplotype: rs1544410, rs2228570, rs2238136, rs4516035, rs11568820, and rs7970314; ii) *DBP* haplotype: rs16846876, rs12512631 and, rs7041; iii) *DHCR7* haplotype: rs12785878, and rs3829251. **Abbreviations**: OR odds ratio; 95% CI, 95% confidence interval; *p*-value, level of significance, *q*-values, *p*-values corrected for multiple testing using the false discovery rate (FDR) with Benjamini and Hochberg procedure.

**Supplemental Figure 1**. Pairwise linkage disequilibrium (LD) patterns. Each diagonal represents a different SNP, with each square representing the coefficient of linkage disequilibrium (D’) or r2 data for pairwise comparison between two SNPs.


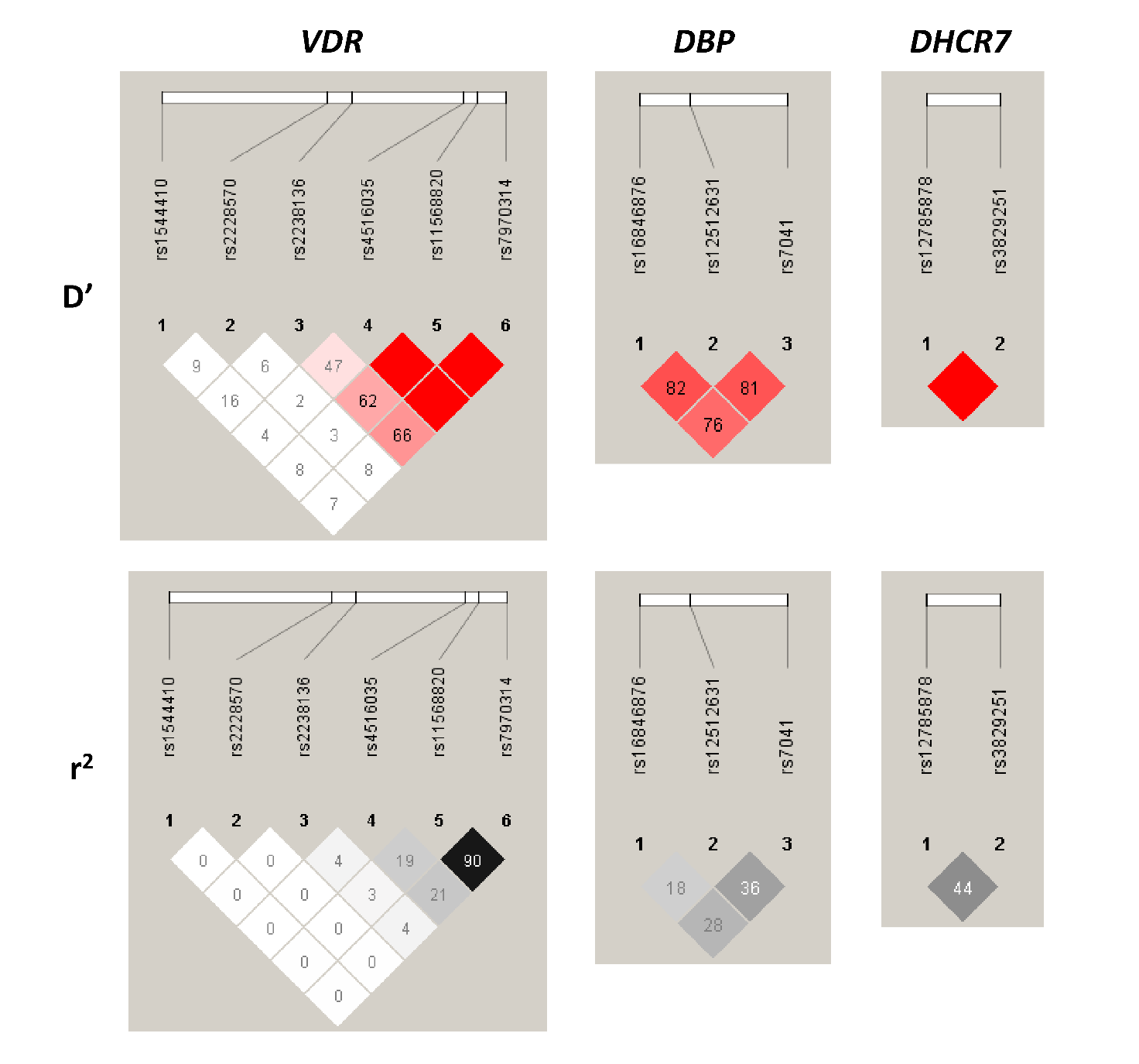

Supplement: Supplementary file 2 [file DataSheet1.docx]
